# Female chimpanzees avoid inbreeding even in the presence of substantial bisexual philopatry

**DOI:** 10.1098/rsos.230967

**Published:** 2024-01-17

**Authors:** Lauren C. White, Veronika Städele, Sebastian Ramirez Amaya, Kevin Langergraber, Linda Vigilant

**Affiliations:** ^1^ Department of Primatology, Max Planck Institute for Evolutionary Anthropology, Leipzig, Germany; ^2^ Arthur Rylah Institute for Environmental Research, Department of Energy, Environment and Climate Action, Melbourne, Australia; ^3^ Institute of Human Origins, School of Human Evolution and Social Change, Arizona State University, Tempe, AZ, USA

**Keywords:** inbreeding avoidance, chimpanzees, exome capture, bisexual philopatry, dispersal, relatedness

## Abstract

Inbreeding (reproduction between relatives) often decreases the fitness of offspring and is thus expected to lead to the evolution of inbreeding avoidance strategies. Chimpanzees (*Pan troglodytes*) are expected to avoid inbreeding as they are long-lived, invest heavily in offspring and may encounter adult, opposite sex kin frequently, especially in populations where both males and females commonly remain in the group in which they were born (bisexual philopatry). However, it is unclear whether substantial bisexual philopatry has been a feature of chimpanzees' evolutionary history or whether it is a result of recent anthropogenic interference, as the only groups for which it has been documented are significantly impacted by human encroachment and experience notable rates of potentially unsustainable inbreeding. Here we use 14 years of observational data and a large genomic dataset of 256 481 loci sequenced from 459 individuals to document dispersal and inbreeding dynamics in an eastern chimpanzee (*P. t. schweinfurthii*) community with low levels of anthropogenic disturbance. We document the first case of substantial bisexual philopatry in a relatively undisturbed chimpanzee community and show that, despite an increased inbreeding risk incurred by females who do not disperse before reaching reproductive age, natal females were still able to avoid producing inbred offspring.

## 1. Introduction

Offspring produced by close relatives often suffer from reduced fitness [1]. This phenomenon, known as inbreeding depression, is primarily caused by the homozygous expression of recessive deleterious alleles [2] and is predicted to lead to the evolution of inbreeding avoidance strategies, such as sex-biased dispersal and kin-recognition [3,4]. However, inclusive fitness theory predicts that inbreeding may be preferred or tolerated at some level as it can be thought of as ‘helping kin to breed’ [5,6]. Additionally, inbreeding avoidance strategies may incur costs to individuals, such as ‘missed opportunity’ costs due to time-consuming screening of potential mates [7]. These conflicting selective pressures are reflected in empirical studies of wild animal populations, which report highly variable results, including inbreeding avoidance [8–11], tolerance [12–14] and even preference for reproducing with relatives [15,16].

Chimpanzees (*Pan troglodytes*) have several characteristics that are likely to favour the evolution of inbreeding avoidance [17]. They reproduce and develop slowly, invest greatly in each offspring [18,19] and their long lifespan can lead to adult, opposite-sex kin encountering each other frequently. Male chimpanzees are strictly philopatric, remaining in their natal groups for their entire lives. By contrast, the percentage of females that remain in the group in which they were born varies across populations (8.3–62.5%, *N* = 7 communities; [20]). Higher rates of bisexual philopatry are expected to lead to greater inbreeding risk through increasing the number of close kin that are members of the same community [21].

However, it is yet unclear whether substantial bisexual philopatry has been a common feature in the evolutionary history of chimpanzees or whether it is a result of recent anthropogenic interference. Thus far, high rates of female philopatry (greater than or equal to 50%) among chimpanzees have only been observed at two substantially human-disturbed sites [22,23]. One of these sites, in Gombe National Park, Tanzania, is an atypically small and isolated population of eastern chimpanzees (*P. t. schweinfurthii*) with limited dispersal options for females due to encroaching human development [20]. Although recent work has indicated the presence of inbreeding avoidance behaviours in female Gombe chimpanzees, inbreeding rates were still notable, with approximately 6% of all offspring surviving at least to the age of two produced by half-sibling or parent–offspring pairs (parental genetic relatedness greater than or equal to 0.25; [24]). This suggests that avoidance behaviours among chimpanzees are not strong enough to totally counteract the increased inbreeding risk caused by high rates of non-dispersing females and further implies that substantial bisexual philopatry may not be sustainable in the long term.

A broader understanding of the variability in dispersal and inbreeding patterns in chimpanzees has been hampered by the difficulty of empirical assessment in wild populations. Because knowledge of pedigree relationships is incomplete, researchers rely upon genetic data to estimate relatedness and evaluate inbreeding risk. Thus far, Gombe is the only chimpanzee population for which dispersal has been conclusively shown to lower inbreeding risk, and within-community inbreeding avoidance has been documented [24]. This work found that adult female chimpanzees who immigrated into their community from a neighbouring one before reproducing had lower average relatedness to the adult males that they co-resided with than females that never dispersed from their natal community, indicating reduced inbreeding risk after dispersal. Furthermore, female chimpanzees at Gombe reproduced with males that were, on average, less related to them than would be expected under random mating, indicating within-community inbreeding avoidance [24]. However, genetic relatedness in this study was assayed with only 4–11 autosomal microsatellites, and calculated on the same set of individuals as those used to estimate population allele frequencies. These factors mean that the estimates of genetic relatedness are expected to not only be imprecise [25,26], but also downwardly biased [27–29]. Only recently has high throughput sequencing technology made it possible to more accurately estimate genetic relatedness from genomic data [30,31], and recent optimizations allow extension to non-invasively collected samples [32,33].

In this study, we investigated dispersal and inbreeding among wild chimpanzees at the Ngogo community in Kibale National Park, Uganda. This large community of eastern chimpanzees has experienced very low levels of anthropogenic disturbance and been the subject of long-term demographic and behavioural data collection [34]. We use 14 years of observational data and a genomic dataset of 256 481 loci sequenced from 212 Ngogo and 247 wider-Kibale chimpanzees to increase our understanding of the variability in dispersal and inbreeding across chimpanzee populations. We first examine the rates of female dispersal and philopatry at Ngogo. Second, we test the expectation that a female's inbreeding risk, as defined by her average relatedness to the pool of potential sires, is lowered by dispersing. Third, we ask whether inbreeding avoidance is occurring within the community by investigating whether the relatedness of actual parent pairs is lower than expected under random mating. In this last test we consider whether females were born in Ngogo (natal) or whether they joined after reaching maturity (immigrants), as these two groups are likely to have different levels of inbreeding risk, and differing mechanisms of kin avoidance. Thus, this study both replicates and improves upon previous work on dispersal and inbreeding in wild eastern chimpanzees.

## 2. Material and methods

### 2.1. Study site and community

Ngogo is a large (138–207 individuals during the study period) community of eastern chimpanzees which occupies a territory of approximately 35 km^2^ in the centre of the mid-altitude rainforest of Kibale National Park, Uganda [35]. Adult males at Ngogo have been identified and monitored since 1995, while females have been identified and regularly followed since 2004. The ages of natal individuals born after monitoring began are known to within 1 year, mostly to within one month [34] and the conception date is calculated as 228 days prior to an individual's estimated birth date [36]. Ages of older individuals are estimated based on their physical appearance, pedigree relationships to other individuals, and/or behaviour [34]. Immigrant females are assumed to be 13 years old when they arrive in Ngogo [34].

The Ngogo pedigree has been constructed based on the analysis of 19–44 microsatellite loci to assign parentage [37]. Older individuals whose parents were not in the community when sampling began, or females who immigrated into the community are pedigree ‘founders’ (*N* = 102 founders in the current pedigree) and are thus assumed to be unrelated to each other when calculating relatedness using the pedigree.

The Ngogo community fissioned into two new groups in 2018. This split occurred gradually, with the first signs noticeable to observers in July 2015 [38]. However, up until January 2018, males and females from the entire community continued to associate and mate. Thus, we consider all members of the Ngogo community together and define our study period as between January 2004 (when females began being regularly followed) and August 2018 (when aggression between the two split Ngogo communities began). The community size over this time increased steadily, peaking in mid-2016 (electronic supplementary material, figure S1). Considering offspring born in this time period, the earliest age at first conception was 10.95 years for males and 11.77 years for females born in Ngogo. We therefore use 10.5 years as a minimum threshold for defining an individual as ‘reproductively aged’.

### 2.2. Rate of female philopatry

We calculated the rate of female philopatry as the percentage of females born in the community who reached the typical age of female dispersal (13 years) during the study period, but remained in the Ngogo community until present day.

### 2.3. Pedigree versus genetic relatedness

Pairwise relatedness calculated from the Ngogo pedigree may be inaccurate because we do not know the relatedness of the pedigree founders, including females who have immigrated into the community. We evaluated the completeness of the Ngogo pedigree and our ability to document inbreeding using pedigree links alone by establishing the pedigree depth (the number of ancestral generations in the pedigree) for all offspring born during the study period. We also documented any inbreeding events captured by the pedigree by establishing whether any parents of offspring born during the study period had known pedigree links.

Pedigree relatedness can also be inaccurate because realized or genetic relatedness (the proportion of genome shared through common descent) will vary from pedigree expectations due to recombination during meiosis. Therefore, to accurately estimate inbreeding risk and avoidance, relatedness should be estimated from genetic data. While microsatellite data were already available for most Ngogo individuals, estimates of relatedness from microsatellites are highly imprecise [39]. Genome-wide single nucleotide polymorphism (SNP) markers, on the other hand, yield accurate estimates of relatedness and can now be generated using high-throughput sequencing [33,39]. We therefore produced a large, genome-wide, SNP dataset using hybridization capture and sequencing of exomes from Ngogo individuals.

### 2.4. Sample collection, extraction and screening

To generate our SNP dataset, we relied mostly on non-invasively collected faecal samples as well as a small number of tissue and bone samples (collected from necroscopies) and urine samples. Samples were collected from habituated Ngogo individuals (between 2011 and 2019) and opportunistically during routine surveys of the wider Kibale National Park for the removal of illegal snares (between 2011 and 2017). These wider-Kibale samples were collected to allow the accurate calculation of population background allele frequencies. Details of DNA extraction and individual identification via microsatellite genotyping are given in electronic supplementary material, methods S1.

The proportion of chimpanzee DNA contained in each DNA extract (hereafter referred to as subject DNA) was estimated using a quantitative polymerase chain reaction (qPCR) assay and Fragment Analyzer (Agilent) quantification following White *et al*. [33]. For non-Ngogo individuals we selected extracts which were estimated to contain greater than 2% subject DNA for library preparation, exome capture and sequencing. For each Ngogo individual we used the extract with the highest estimated proportion subject DNA, occasionally processing extracts from multiple samples per individual when low concentrations made this necessary.

The data presented in this study include exome sequence data from 112 individuals that were first sequenced as part of a previous study [33]. Here we augment the data for 102 of those 112 individuals to increase depth of coverage.

### 2.5. Library preparation and shotgun sequencing

Each of the chosen extracts was converted into up to four separate libraries depending on the estimated proportion of subject DNA. Extracts with greater than 2% subject DNA were converted into one library, those with 1–2% were converted into two libraries, those with 0.5–1% were converted into three libraries, and the small number with less than 0.5% were converted into four libraries.

Library preparation followed the methods described by White *et al*. [33]. Briefly, after shearing via ultrasonication using the Covaris S2 (Covaris) or the Biorupter UCD-200 (Diagenode), extracts were converted to sequencing libraries following a modified ‘BEST’ method [40], which is an optimized version of the widely used blunt-end ligation method [41]. After library preparations, two PCRs were performed in parallel on the nick-repaired libraries. One PCR prepared the libraries for shotgun sequencing by extending the Illumina adapter sequence to full-length, while the other PCR prepared libraries for hybridization capture and thus did not extend the adapter sequence. Shotgun-prepared libraries (including no-template control libraries) were combined into four pools in equi-molar concentrations. Since no-template controls had measured concentrations of zero, we simply added the 2 µl (the average volume added per extract library) of each control to the pools.

Each pool was then sequenced on one lane of an Illumina HiSeq 2500 in rapid mode (125 bp read lengths, paired-end). Raw shotgun reads were de-multiplexed and internal barcodes removed using sabre (https://github.com/najoshi/sabre). Adapter contamination was trimmed using bbduk (part of the bbtools software suite: sourceforge.net/projects/bbmap/), before we mapped reads to the chimpanzee reference genome (panTro6; [42]) using bwa-mem (v. 0.7; [43]) with default settings. Duplicate reads were identified and marked using PicardTools (v. 2.27.4 http://broadinstitute.github.io/picard/). Mapped reads with a quality score of less than 30, or that were less than 35 bp long, were excluded using Biohazard-Tools (https://github.com/mpieva/biohazard-tools). The shotgun sequencing results were used to re-estimate the proportion of subject DNA for each library as the proportion of sequenced reads that mapped to the chimpanzee genome and passed filtering [33].

### 2.6. Hybridization capture and sequencing

Prior to hybridization we used the shotgun estimates of proportion of subject DNA to pool libraries in equi-endogenous ratios. Libraries were combined into 115 pools of 2–10 libraries, 12 of which were captured and sequenced as part of an earlier study [33]. If the concentration allowed, some libraries were captured in multiple pools.

Hybridization capture was performed as described in White *et al*. [33]. We captured all library pools using the SureSelect XT Human All Exon V6 RNA Library baits (target space approximately 60 Mb, Agilent). We followed the manufacturer's instructions but used home-made buffers and custom Xgen blocking oligos (IDT).

We combined capture pools in equi-molar ratios into four sequencing pools and sequenced each on two lanes of the Illumina HiSeq 2500 system in rapid mode (125 bp read lengths, paired-end). Reads were processed and mapped as above, and the resulting bam files were used to predict the likely gain in unique reads from increased sequencing effort using the *lc_extrap* option of the program PRESEQ (v. 3.1.2; [44]). PRESEQ results were corrected using the library-specific exome mapping rate as in White *et al*. [33]. This shallow round of sequencing and PRESEQ analysis enabled us to estimate the complexity of each library and ensure that we did not waste resources in over-sequencing. Using the PRESEQ results to set ‘goal’ sequencing efforts per library, we repooled capture pools in appropriate ratios and sequenced them deeply using seven flow cells on the Illumina HiSeq 2500 system in high-output mode (125 bp read lengths, paired-end).

We de-multiplexed, adapter trimmed, mapped and filtered our sequencing data as above. Libraries sequenced across multiple lanes were merged using samtools (v. 1.11, [45]) before identifying and removing duplicate reads.

### 2.7. Quality checks and filtering

Chimpanzees hunt and eat other primate species [46]. Thus, it is possible that non-target primate DNA present in some extracts may be co-captured during hybridization and impact downstream analyses. We therefore used multiple approaches to detect potential contamination in our sequencing data. Briefly, we used metagenomic read classification (Kraken2, v. 2.0.8-beta; [47]) and heterozygosity to conservatively exclude potentially contaminated libraries and samples. We furthermore examined preliminary genetic relatedness estimates for deviations from expected relatedness based on the known pedigree to check for cross-contamination, mislabelling and errors in assigned pedigree relationships. Further details are in the electronic supplementary material, methods S2 and S3, table S1 and figures S2 and S3.

After merging libraries per individual as above, depth of coverage of the target region was calculated using bedtools *coverageBed* (v. 2.30.0; [48])*.* The target region includes the exome regions covered by the SureSelect baits (approximately 60 Mb or 1.5% of the chimpanzee genome). To convert the coordinates of the target regions (as provided by Agilent) from hg19 to panTro6 we used the liftover utility from UCSC with default parameters [49]. As suggested by the authors of ngsRelate (v. 2, [50,51]), we excluded 23 individuals (19 Ngogo individuals and four individuals from wider Kibale) with an average depth of coverage of less than 4× from all downstream analyses.

### 2.8. Variant detection and genotype likelihood estimation

To ascertain a high-quality SNP set, we identified variant sites from individuals with medium to high average depth of coverage (greater than 10×) and whose samples were collected opportunistically from the Kibale-wide area during snare patrols (*n* = 250). We refer to this subset as the ‘high-quality sample subset’. The depth of coverage filter was used to reduce the chance of erroneous variant sites being included in our SNP set, and the particular method of collection gives us greater confidence that these samples are a representative random sample of the wider Kibale chimpanzee population. This is crucial to enable accurate estimation of population allele frequencies, which is key for our final relatedness estimates, as well as for further filtering of our SNP set based on Hardy–Weinberg equilibrium, minor allele frequencies (MAF) and linkage disequilibrium. Using this subset for SNP identification and allele frequency estimates also circumvents problems that arise when a disproportionate number of close kin (relative to their distribution in the population) are included in allele frequency estimation [29], which would have been the case had we used our entire dataset in which Ngogo individuals spanning multiple generations and intertwined pedigrees were prevalent. Finally, the subset also allows us to avoid issues that may arise when close kin are purged from datasets, which can potentially lead to under-representing kinship relative to the population [52], as we assume close kin have been collected at a rate that is proportional to their distribution across the population.

We ran ANGSD (v. 0.925; [53]) using the high-quality sample subset to identify variant sites, and calculate minor allele frequencies (-SNP_pval 1e-6, -doGLF 3, -doMajorMinor 1 -doCount 1 -doMaf 1). We excluded reads with multiple hits that were not the primary read (-uniqueOnly 1 -remove_bads 1) and with map quality scores below 30 (-minMapQ 30) after adjusting for excessive mismatches (-C 50). We also excluded sites with quality scores below 20 (-minQ 20), more than two alleles (-rm_Triallelic) and depth of coverage below 3 and above 200 (-setMinDepth 3, -setMaxDepth 200). Finally, we required that sites had data in at least 225 individuals (90% of our high-quality sample subset; -minInd 225), that the total number of reads per site across individuals was at least 675 (roughly 3 reads per individual; -minDepth 675) and that sites were in Hardy–Weinberg equilibrium (-doHWE -HWE_pval 0.001). We ran each autosome separately (-r) and merged the results for downstream analyses. We then used ANGSD to estimate genotype likelihoods for all SNPs identified in the above steps for our entire dataset (-GL 2 -doGLF 3 -doMajorMinor 3 -sites).

### 2.9. Genetic relatedness estimation

We used ngsRelate (v. 2, [50,51]) and the Hedrick and Lacy [54] method to estimate pairwise genetic relatedness between all Ngogo individuals in our final SNP dataset using allele frequencies estimated from the high-quality subset. We chose the Hedrick and Lacy [54] method as it considers all possible patterns of identity-by-descent sharing between two individuals to account for potential inbreeding, and the implementation in ngsRelate allows genotype likelihoods to be used. We validated our genetic relatedness estimates by calculating the correlation (using the *lm* function in R) between the ngsRelate SNP relatedness estimates and the expected relatedness (calculated using the path counting method; [55]) between pairs of known relatives extracted from the Ngogo pedigree. We also used this validation method to examine the impact of removing linked SNPs and using allele frequencies calculated from the entire dataset (rather than the high-quality subset) on relatedness estimates (presented in the electronic supplementary material, methods S4 and figure S4).

### 2.10. Inbreeding risk and avoidance

To investigate inbreeding risk and avoidance in female chimpanzees at Ngogo, we examined mother–male genetic relatedness as a function of a female's natality status and whether the pair were *actual* or *potential* parents. We defined actual and potential parents for all offspring born during the study period and for whom both parents are known (through observation or microsatellite analysis) and included in our SNP dataset. *Actual* parents are the known parents of each offspring. *Potential* parents were defined as the mothers of each offspring, paired with all other adult males (included in the SNP dataset) that co-resided with that mother within a month (±15 days) of the conception date of the offspring in question. Thus, potential parent-pairs represent the expected relatedness of parents under random mating. Females who could not be classified as either ‘natal’ or ‘immigrant’ because they were not observed migrating into the community (i.e. individuals who were already in the community when observations began) and their parents have not been identified within Ngogo, are considered to have ‘unknown’ natality status. These represent the oldest mothers at Ngogo, who are expected to be combination of natal and immigrant females (electronic supplementary material, figure S5).

We used a beta regression generalized linear mixed model (GLMM) because our outcome variable, genetic relatedness as estimated from our SNP dataset, is bounded between zero and one [56]. As extreme boundary values (zero and one) are not defined in logit-link beta distributions, and our observations include zeroes, we added 0.0001 to all observed genetic relatedness values for identifiability of model parameters [57]. The categorical predictor variables were whether the pair were actual or potential parents, the female's natality status (natal, immigrant, or unknown), and the interaction between these predictors.

As our observations include some mothers and sires who had multiple offspring within the study period, and both individuals within each pair (female and male) are represented across multiple observations, we included varying effects for each offspring (to account for mothers/sires with multiple offspring) and each individual within a pair (to account for non-independence of the pairwise relatedness outcome variable). Individual varying effects were included using a joint indexing notation as described by [58], which allowed us to accurately pool individual-level information across dyads. To improve model efficiency, we used a non-centred parametrization by applying a Cholesky decomposition to the varying effects priors [59].

The GLMM was fit using the Hamiltonian Markov chain Monte Carlo (MCMC) engine R-Stan (v. 2.19.2; [60]) implemented using the *map2stan* function of the *rethinkin*g package (v. 2.0; [59]) in R (v. 3.5.3; [61]). We used weakly regularizing priors to ensure that our model was sceptical of impossibly extreme relationships, and examined trace plots, the effective sample size and $\hat{R}$ values after model fitting to ensure convergence and sampling efficiency. We present our model results as median posterior predictions, 89% credibility intervals [59] and density plots of 1000 predictions for each combination of categorical predictors drawn from the posterior. Model code and input data are available at https://github.com/DrLaurenCWhite/Chimpanzee_Inbreeding_Avoidance.

## 3. Results

### 3.1. Sequencing results

We estimated the proportion of chimpanzee DNA in 3496 extracts (including 3441 faecal, 41 urine, three bone and 11 tissue extracts) and chose 555 for sequencing (including 536 faecal, 12 urine, two bone and five tissue extracts). These were converted into 800 libraries representing 495 individuals. We shotgun-sequenced an average of 1 592 363 reads (s.d. = 66 388.7) per library and, after demultiplexing and mapping of shotgun data, found that these contained, on average, 6.4% subject DNA (s.d. = 9.7%, a breakdown by sample type is given in electronic supplementary material, table S2). None of our 33 no-template controls had reads mapping to the chimpanzee genome. After hybridization capture, we sequenced an average of 31 973 407 reads per captured library (s.d. = 17 908 923), and found that the average proportion of reads mapping to the chimpanzee genome had increased to 60.4% (s.d. = 24.5%, a breakdown by sample type is given in electronic supplementary material, table S2).

After SNP identification and filtering, our final dataset consisted of 256 481 autosomal SNPs. Of the 36 individuals removed from the final set, 23 were excluded due to low coverage, nine were excluded due to potential cross-species contamination (electronic supplementary material, methods S2, table S1 and figures S2 and S3), and four were excluded due to potential inter-individual contamination (electronic supplementary material, methods S3). The final dataset of 459 individuals includes 212 Ngogo individuals (92 male and 120 female, mean depth of coverage = 16.2×, s.d. = 9.5) and 247 unhabituated individuals belonging to other communities in the park (134 male and 113 female, mean depth of coverage = 23×, s.d. = 13). This dataset comprises between 56% and 94% of the known reproductively aged Ngogo community during our study period (2004–2018). The proportion of the reproductively aged community that is included in the SNP dataset increases over time as individuals who were alive and in the community after screening of extracts began (mid-2017) could be resampled to increase the chance that a sample with adequate subject DNA would be collected (electronic supplementary material, figure S1). By contrast, individuals who had died or otherwise left the community could not be resampled. Nevertheless, at any particular time point within the study period, we consider our dataset a representative sample of the reproductively aged Ngogo community at that time.

### 3.2. The rate of female philopatry

During our study period, 18 of the 36 females (50%) that were born in Ngogo and reached the typical age at which female chimpanzees disperse to join a new community (13 years; [34]) instead remained at Ngogo until present day. The females who emigrated from Ngogo during our study period did so at an average age of 12.7 years (range: 9.2–16.1 years; electronic supplementary material, figure S6). By contrast, by the end of the study period, the non-emigrating natal females had lived an average of 8.5 years in Ngogo since reaching age 13 (range: 0.2–20 years; electronic supplementary material, figure S7) and 15 of these females had reproduced. During the study period non-emigrating natal Ngogo females produced 21 offspring sired by Ngogo males (there were no known extra-group paternities).

### 3.3. Inbreeding documented by the current pedigree

Of the total 159 offspring born during the study period, 114 had known parentage through previous microsatellite genotyping. The remaining 45 offspring were unable to be checked for paternity as samples had either not yet been collected (i.e. those born late in the study period), or the offspring had died before sampling could occur. Of the 114 offspring with known parentage, the Ngogo pedigree revealed that one (0.9%) was produced by a natal female and her paternal half-nephew (pedigree-based *R* = 0.125). No other parent pairs were linked via common ancestry on the pedigree. However, 98 of the offspring for whom both parents were known have a pedigree depth of only one, meaning that one or more of their grandparents are unknown. Thus, the pedigree is not sufficiently deep for accurate assessments of relatedness of the parental pairs. We therefore used our new estimates of genetic relatedness to reveal any cryptic inbreeding events.

### 3.4. Parental relatedness estimated using the single nucleotide polymorphism dataset

**Figure 1.**
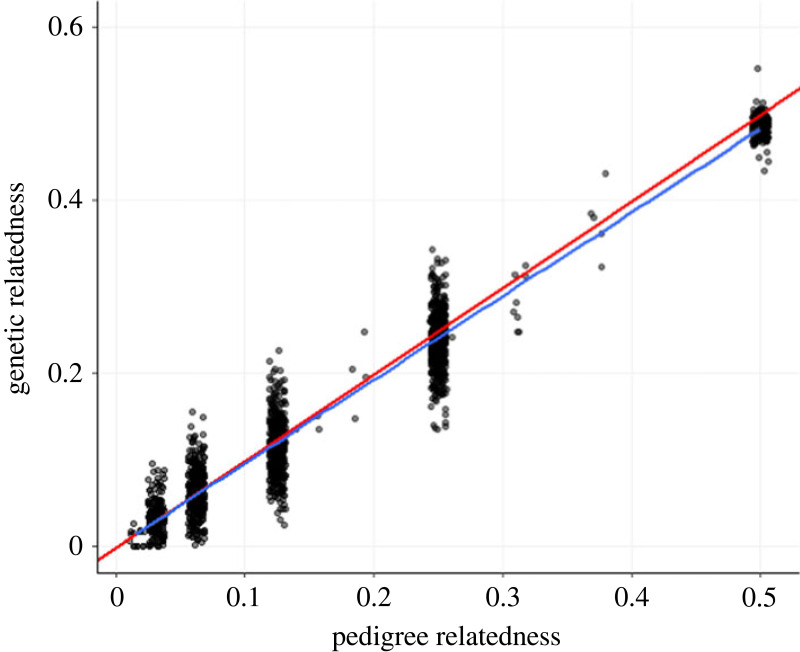
Validation of genetic relatedness estimates (*y* axis) by comparison with expected relatedness calculated from the Ngogo pedigree (*x* axis). Points represent pairs of Ngogo individuals (*n* = 1615), the red line represents a 1:1 correlation, the blue line represents the observed correlation estimated via linear regression (*R*^2^ = 0.96, *p* < 0.001).

Our estimates of genetic relatedness were highly correlated with the expected average relatedness as calculated from the Ngogo pedigree (*R*^2^ = 0.96, *p* < 0.001; figure 1) and the strength of this relationship was maximized when using allele frequencies calculated from the high-quality sample subset, and a SNP set that was not pruned for linkage (electronic supplementary material, figure S4). As expected, there was substantial variation in genetic relatedness around the means (figure 1).

**Figure 2.**
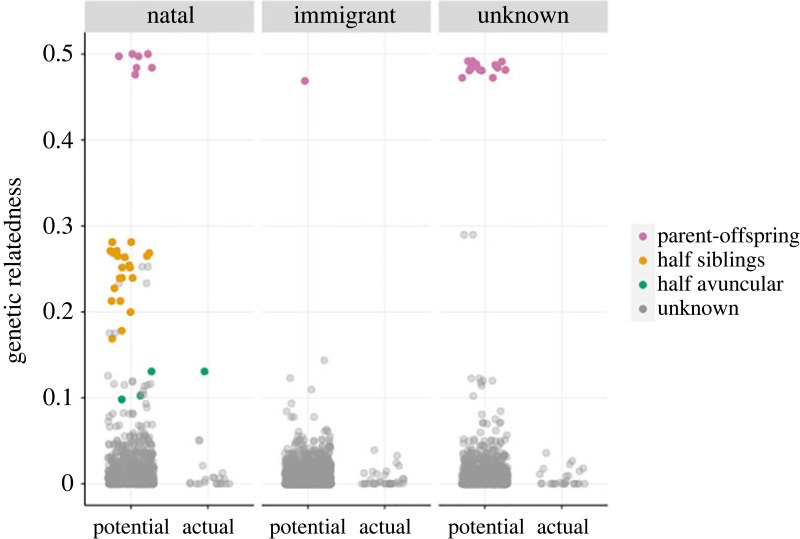
Genetic relatedness of actual and potential parent pairs including mothers with different natality status (natal, immigrant or unknown natality) and males that were actual or potential sires of offspring. Each point represents a mother–male pair (for which both individuals are reproductively aged and included in our SNP dataset) and colours represent pedigree relationship category (if known).

Of the 114 offspring with known parentage, 79 had both their mother and father included in our SNP dataset, enabling us to estimate genetic relatedness of their parents. Among these 79 parental pairs, we did not find any with genetic relatedness larger than 0.05 other than the known inbreeding event mentioned above; figure 2. We did, however, identify 178 non-parent pairs of Ngogo individuals who are unconnected on the current pedigree, but whose genetic relatedness is in the range of 0.1–0.35, indicating likely recent, unaccounted for, pedigree links (electronic supplementary material, figure S8). This group includes 16 pairs of individuals who are assumed pedigree founders (i.e. for whom no parental links are assigned), 14 of which are pairs of immigrant females or females with unknown natality (electronic supplementary material, figure S8). Importantly for our analysis of inbreeding avoidance, we also identified 32 mother–male potential parent pairs with genetic relatedness in the range of 0.1–0.35 (figure 2), which would have otherwise been assigned a relatedness value of zero, if we had relied solely on the current pedigree. These cryptic relationships, and the high variance in genetic relatedness found for all pedigree relational categories (excepting parent–offspring, figure 2), reinforce our assertion that the Ngogo pedigree alone could not have provided accurate estimates of relatedness for our analysis of inbreeding risk and avoidance.

### 3.5. Inbreeding risk and avoidance

Our modelled data included 2805 mother–male pairs, of which 79 were actual mother–sire pairs (representing the parents of 79 offspring) and 2726 were potential mother–sire pairs (figure 2). The individuals included in this dataset include 45 mothers and 52 males. Of the included males, 30 were actual sires in at least one of the 79 actual mother–sire pairs, with the remaining 22 males included only as potential sires. Of the 45 mothers, 12 were natal, 20 were immigrants and 13 had unknown natality. A breakdown of the number of mother–male pairs for each combination of female natality and actual/potential sire is given in table 1.

**Table 1.** Model sample sizes (*N*) and posterior predictions.

natality status of the female parent	paired with…	*N*	median posterior predicted relatedness	89% credibility interval	difference in predicted relatedness (actual versus potential sires)
natal	actual sires	21	0.015	0.009–0.02	−34.78%
	potential sires	738	0.023	0.021–0.026	
immigrant	actual sires	33	0.013	0.01–0.018	−18.75%
	potential sires	1221	0.016	0.015–0.018	
unknown	actual sires	25	0.013	0.009–0.019	−31.58%
	potential sires	767	0.019	0.016–0.022	

**Figure 3.**
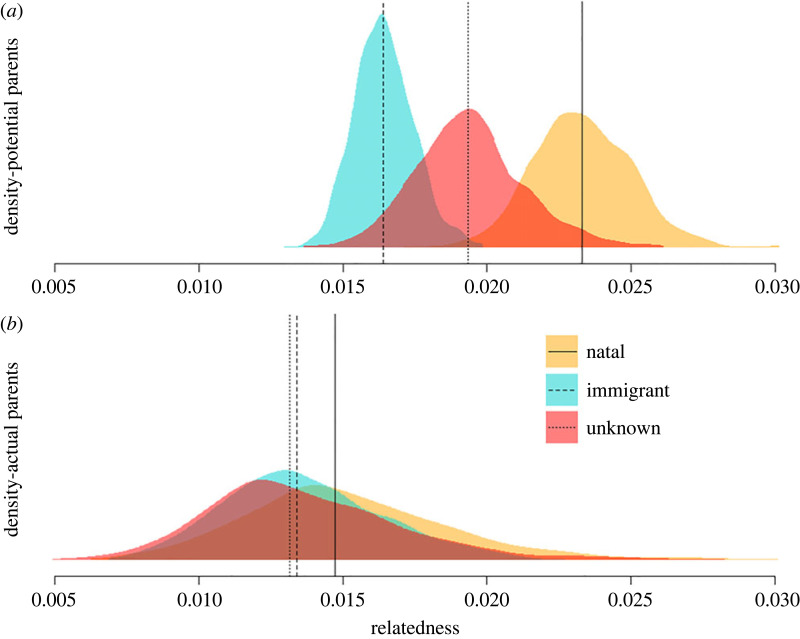
Inbreeding risk and avoidance model results: density plots of 1000 posterior predictions of average genetic relatedness between mother–male pairs for each combination of categorical predictors (actual/potential parents and female natality status). (*a*) ‘*Potential’* parents were defined as the mother of each offspring, paired with all other reproductive-aged males that resided in the community within ±15 days of the estimated conception date of the offspring in question. (*b*) ‘*Actual*’ parents are the known parents of all offspring conceived during the study period. Vertical lines represent the posterior median predictions. *N* = 79 actual and 2726 potential parent-pairs. The model excluded females who did not reproduce during the study period, and individuals not included in our SNP dataset. The same results, separated by natality status, are shown in electronic supplementary material, figure S9.

Model posterior predictions and 89% credibility intervals are shown in table 1, figure 3 and electronic supplementary material, figure S9, while posterior coefficient values are given in electronic supplementary material, table S3. The average relatedness of natal mothers to the pool of potential sires was 30% greater than the immigrant mothers and 17% greater than the mothers of unknown natality, suggesting that dispersal lowers inbreeding risk. Nevertheless, natal mothers were no more related to the actual sires of their offspring than were immigrant or unknown natality mothers. Our model suggests that this is due to successful inbreeding avoidance by natal females who were, on average, 35% less related to the sires of their offspring than to other males in the community (i.e. 35% less related to sires than expected under random mating). Immigrant and unknown mothers were also 19 and 32% less related, respectively, to the sires of their offspring than expected under random mating. However, there was considerable uncertainty in this result (i.e. moderate overlap in posterior distributions and credibility intervals between actual and potential parent categories for immigrant and unknown females; figure 3, table 1).

## 4. Discussion

Our results show that substantial levels of bisexual philopatry among chimpanzees are not only observed in small, isolated populations with high levels of human disturbance. Ngogo is a large community with numerous neighbouring communities surrounding it on all sides. Thus, the substantial proportion of non-dispersing females (50%) cannot be attributable to a lack of dispersal opportunities due to isolation, community size or human disturbance. Furthermore, although immigrant females were at lower risk of inbreeding, the females who chose not to disperse were still capable of avoiding breeding with their male relatives within the community. This suggests that substantial bisexual philopatry may be a feature of chimpanzee evolutionary history, as it does not necessarily lead to close inbreeding and its potential negative fitness consequences.

The results of our inbreeding risk and avoidance analysis based on SNP-derived estimates of genetic relatedness are similar to those of Walker *et al*. [24] from the Gombe population which were based on lower resolution microsatellite data. This confirms the expectation that, in chimpanzees, inbreeding risk may be lowered through female-biased dispersal and supports the hypothesis that inbreeding avoidance is also occurring within chimpanzee communities. Our results found the strongest evidence for within-community inbreeding avoidance among natal females, who are expected to have the most male relatives within the community, and the potential to recognize kin through familiarity-based mechanisms.

We also found weak evidence of within-community inbreeding avoidance by immigrant females and females with unknown natality. This concurs with the results of Walker *et al*. [24] at Gombe, where inbreeding avoidance by immigrant females was also shown. Our examination of cryptic relatedness revealed numerous pairs, including pairs in which one or both individuals were an immigrant female or female with unknown natality, with genetic relatedness within the range expected for full-cousins to half-siblings. This suggests that even recently immigrated females may have male relatives in the community with whom it would be beneficial to avoid breeding. Mechanisms to avoid unfamiliar kin could include phenotype-matching [62] or sperm selection [63]. For example, a recent study of captive chimpanzees found possible group- and kin-recognition via olfactory cues [64]. However, our results on inbreeding avoidance by non-natal females lacked strong support in our model, possibly because dispersal had effectively lowered the inbreeding risk of immigrant females to a point where further inbreeding avoidance was unnecessary and/or undetectable in our dataset. The intermediate results for females of unknown natality status are expected if these individuals are a combination of natal and immigrant females, as we suspect.

Our results lead to the question of why a female chimpanzee would choose to disperse at all. Compared with chimpanzee females who remain in their natal community to breed, immigrant females leave familiar social contexts and environments, experience elevated stress and female–female aggression and begin producing offspring 2.5 years later than non-dispersing females [19]. Thus, dispersal is risky, and we should expect strong benefits to overcome these costs. What we found, however, was that while the reduction in inbreeding risk from dispersing was statistically detectable, average relatedness of reproductively-aged mother–male pairs (and thus inbreeding risk) was quantitatively low overall, even among natal females (i.e. average genetic relatedness less than 0.03, figure 3). Furthermore, had the immigrant females remained in their natal community, our results suggest they would have still been capable of avoiding breeding with their male kin.

To ascertain the true influence of inbreeding risk on facultative dispersal, it should be investigated with the full range of possible proximate causes, including intra-sexual competition, kin support, kin competition and resource availability [65,66]. For example, dispersal in the Kanyawara chimpanzee community was associated with periods of high fruit consumption, supporting the hypothesis that energic conditions impact emigration timing [67]. Furthermore, a recent paper examined factors influencing dispersal at Gombe and found that inbreeding risk and maternal support had opposing effects on a female's likelihood to disperse [22]. A similar examination of these and other factors that may potentially impact dispersal (for example the quality of the habitat, size of the community, or number of kin within the community) at Ngogo would be invaluable to our understanding of dispersal dynamics when inbreeding risk is low overall.

Understanding the influence of inbreeding risk on dispersal in chimpanzees would also profit from an estimation of the strength of inbreeding depression in this species and population. The likelihood of experiencing inbreeding depression increases with the degree of parental relatedness, but there is substantial variation in this relationship and the overall strength of inbreeding depression across taxa, populations and environments [68,69]. For example, island foxes (*Urocyon littoralis*) do not suffer inbreeding depression due to effective purging of recessive deleterious alleles [70]. Conversely, inbreeding depression was detected in an island population of feral horses (*Equus ferus caballus*), but its intensity varied across life stages and ecological contexts [71]. As yet there is only anecdotal evidence for higher mortality of inbred chimpanzee offspring [24,72,73]. Future studies could use the newly generated genomic data to test for inbreeding depression and quantify the genetic load in Ngogo chimpanzees. For example, estimates of genomic inbreeding (such as runs of homozygosity and genome-wide heterozygosity) could be correlated with survival and reproductive success [74] or the prevalence of putatively deleterious variants (e.g. loss-of-function mutations) could be assessed across the genome ([75]; but see [76] for discussion on the difficulties of translating genomic load estimates into realized fitness consequences).

The inability to account for inbreeding depression that lowers the survival of individuals below the age of sampling is a common caveat across all studies of inbreeding avoidance. At Ngogo, genetic samples for paternity assignment are typically taken from infant chimpanzees when they are approximately 2 years old, and so inbred individuals would be missing from our dataset if increased mortality due to inbreeding depression occurs prior to this age. While this issue can be partially resolved by sampling closer to the birth dates of individuals, it cannot be entirely removed as in-utero inbreeding depression cannot be ruled out. Future studies should endeavour to take samples from infants as early as possible, and also document mating rates and interbirth intervals to better understand these dynamics.

In a broader perspective, our results indicate that flexible female-biased dispersal and other forms of inbreeding avoidance may have been common features in the evolutionary history of both *Pan* and *Homo* social structures. Among humans, the strength of bisexual philopatry is extremely varied [77,78] and yet inbreeding between close relatives is rare, occurring mostly in small, isolated populations [79,80]. Inbreeding between close human relatives can be easily avoided (given a large enough population such that unrelated mates are available) because most reproduction occurs in the context of long-term pair bonds, and codification of kinship classes through language allows relatives to easily identify each other [81]. However, our results suggest that inbreeding avoidance in the presence of variable bisexual philopatry has been a characteristic of hominid social structure prior to the emergence of language and pair bonding. Thus, this study contributes to an emerging picture revealing that flexible patterns of dispersal and reproduction are features not limited to the human species, but may have been present deep into our evolutionary past.

## Data Availability

All demultiplexed exome sequencing data is available at NCBI's sequence read archive (SRA) under the accession code PRJNA505752. A list of program versions used for processing raw sequencing data, quality checking results and estimating relatedness is provided in the electronic supplementary material (electronic supplementary material, table S4) [82]. Data and relevant code for this research work are stored in GitHub: https://github.com/DrLaurenCWhite/Chimpanzee_Inbreeding_Avoidance, and have been archived within the Zenodo repository: https://zenodo.org/records/10032093 [83].
